# Region-specific blood–brain barrier transporter changes leads to increased sensitivity to amisulpride in Alzheimer’s disease

**DOI:** 10.1186/s12987-019-0158-1

**Published:** 2019-12-17

**Authors:** Gayathri Nair Sekhar, Alice L. Fleckney, Sevda Tomova Boyanova, Huzefa Rupawala, Rachel Lo, Hao Wang, Doaa B. Farag, Khondaker Miraz Rahman, Martin Broadstock, Suzanne Reeves, Sarah Ann Thomas

**Affiliations:** 1 Faculty of Life Sciences and Medicine, School of Cancer and Pharmaceutical Sciences, King’s College London, Franklin-Wilkins Building, 150 Stamford Street, Waterloo, London, SE1 9NH UK; 2 Faculty of Pharmacy, Misr International University, Cairo, 11431 Egypt; 3 Wolfson Centre for Age-Related Diseases, King’s College London, Guy’s Campus, London, SE1 1UL UK; 4 Maurice Wohl Clinical Neuroscience Institute, King’s College London, 125 Coldharbour Lane, Camberwell, London, SE5 9N UK; 5 Division of Psychiatry, Faculty of Brain Sciences, University College London, 149 Tottenham Court Road, London, W1T 7NF UK

**Keywords:** Amisulpride, MATE1, PMAT, OCT1, Blood–brain barrier, Alzheimer’s

## Abstract

**Background:**

Research into amisulpride use in Alzheimer’s disease (AD) implicates blood–brain barrier (BBB) dysfunction in antipsychotic sensitivity. Research into BBB transporters has been mainly directed towards the ABC superfamily, however, solute carrier (SLC) function in AD has not been widely studied. This study tests the hypothesis that transporters for organic cations contribute to the BBB delivery of the antipsychotics (amisulpride and haloperidol) and is disrupted in AD.

**Methods:**

The accumulation of [^3^H]amisulpride (3.7–7.7 nM) and [^3^H]haloperidol (10 nM) in human (hCMEC/D3) and mouse (bEnd.3) brain endothelial cell lines was explored. Computational approaches examined molecular level interactions of both drugs with the SLC transporters [organic cation transporter 1 (OCT1), plasma membrane monoamine transporter (PMAT) and multi-drug and toxic compound extrusion proteins (MATE1)] and amisulpride with the ABC transporter (P-glycoprotein). The distribution of [^3^H]amisulpride in wildtype and 3×transgenic AD mice was examined using in situ brain perfusion experiments. Western blots determined transporter expression in mouse and human brain capillaries .

**Results:**

In vitro BBB and in silico transporter studies indicated that [^3^H]amisulpride and [^3^H]haloperidol were transported by the influx transporter, OCT1, and efflux transporters MATE1 and PMAT. Amisulpride did not have a strong interaction with OCTN1, OCTN2, P-gp, BCRP or MRP and could not be described as a substrate for these transporters. Amisulpride brain uptake was increased in AD mice compared to wildtype mice, but vascular space was unaffected. There were no measurable changes in the expression of MATE1, MATE2, PMAT OCT1, OCT2, OCT3, OCTN1, OCTN2 and P-gp in capillaries isolated from whole brain homogenates from the AD mice compared to wildtype mice. Although, PMAT and MATE1 expression was reduced in capillaries obtained from specific human brain regions (i.e. putamen and caudate) from AD cases (Braak stage V–VI) compared to age matched controls (Braak stage 0–II).

**Conclusions:**

Together our research indicates that the increased sensitivity of individuals with Alzheimer’s to amisulpride is related to previously unreported changes in function and expression of SLC transporters at the BBB (in particular PMAT and MATE1). Dose adjustments may be required for drugs that are substrates of these transporters when prescribing for individuals with AD.

## Background

Antipsychotic drugs are associated with significant harm in older people, particularly those with dementia who are more susceptible to antipsychotic drug related morbidity (parkinsonism, postural hypotension, stroke) and mortality than other diagnostic groups [1, 2]. This has led to restrictions on the national health service (NHS) use of this class of drugs in the pharmacological management of psychosis and agitation in dementia. Emerging evidence from research into amisulpride use in older people with Alzheimer’s disease (AD) psychosis suggests that blood–brain barrier (BBB) dysfunction may be an important contributor to this heightened sensitivity [3, 4].

Amisulpride is a benzamide derivative, second generation antipsychotic drug, used to treat schizophrenia [5] and a drug for which the optimal dose (400–800 mg/day), blood concentration (100–319 ng/ml) and striatal dopamine D2/3 receptor occupancy range to avoid non-response and parkinsonism (40–70%) are well established in young adults with schizophrenia [6–8]. Despite being highly selective for dopamine D2/3 receptors [in vitro (K_i_ = 2.8 nM) and D3 (K_i_ = 3.2 nM)] amisulpride has a low propensity to induce parkinsonism, due to its poor BBB penetration and mesolimbic selectivity [9]. In an open treatment study which used amisulpride use in older people with AD psychosis and very late-onset (> 60 years) schizophrenia-like psychosis (VLOSLP), treatment response and parkinsonism occurred at very low doses (25–75 mg/day AD, 50–100 mg/day VLOSLP), and at correspondingly low blood drug concentrations (40–100 ng/ml AD, 40–169 ng/ml VLOSLP) due to higher than anticipated striatal dopamine D2/3 receptor occupancies (caudate occupancy, steady state treatment, 50 mg/day amisulpride; 41–83% AD, 41–59% VLOSLP) [3, 10–12]. These findings strongly implicate age and AD-specific changes in central pharmacokinetics in antipsychotic drug sensitivity, particularly at the BBB, which controls drug entry through the expression of transporters [13]. Furthermore, they suggest that amisulpride [14, 15] is a sufficiently sensitive tool with which to probe BBB functionality.

The majority of research into BBB transporters has been directed towards the ABC superfamily, which are ATP-dependent efflux transporters such as P-glycoprotein (P-gp) [16], whose action is compromised in age [17], and more markedly so in AD [18–21]. It has been suggested that amisulpride is a weak P-gp substrate [22, 23], but the importance of P-gp relative to other transporters, especially members of the SLC superfamily, remains unclear. Amisulpride is predominately positively charged (98.9%) at physiological pH (pKa 9.37), and is likely a substrate for the organic cation transporters (OCT) and organic cation transporters novel (OCTN); as observed using the immortalized human cerebral microvessel endothelial cell line (hCMEC/D3) [14]. However, it is also possible that other SLC transporters of organic cations, such as plasma membrane monoamine transporter (PMAT) and multi-drug and toxic compound extrusion proteins (MATEs), are involved. Haloperidol, a first generation antipsychotic drug which is highly selective for D2 receptors [24], was chosen as a clinically relevant comparator, as it is a positively charged molecule at pH7.4 and a OCT1 substrate and inhibitor [25, 26] and likely to utilize similar transporters to amisulpride to cross the BBB. Although the drug is no longer used as a first line treatment for psychosis, due to its propensity to cause parkinsonism, it is used at very low doses in the treatment of delirium, which most commonly occurs in those aged over 65 years and with an underlying cognitive impairment (https://www.nice.org.uk/, accessed 15.11.2019). The drug is also extensively used in palliative care and, as a result, is one of the 20 drugs on the WHO list of essential medications.

This study tested the hypothesis that there was an interaction between amisulpride and influx and/or efflux transporters at the BBB which was relevant from a pharmacodynamic perspective by:

1. identifying the transporter involved in the CNS distribution of amisulpride and haloperidol, by examining their kinetic characteristics and inhibitor sensitivity at the human and mouse BBB in vitro.
2. confirming the molecular level interactions of amisulpride and haloperidol with the selected BBB transporters using an in silico computational approach.
3. establishing whether amisulpride access to the CNS is increased in transgenic AD mice, which harbour the human amyloid precursor protein (APP)-Swedish mutation (KM670/671NL), tau mutation (P301L), and presenilin-1 mutation (M146V) compared to wildtype mice.
4. investigating transporter expression in human (and mouse) brain endothelium from age-matched post-mortem AD and healthy aged controls.
5. Examining the type of medications prescribed to individuals with AD and age matched controls.

**Fig. 1:**
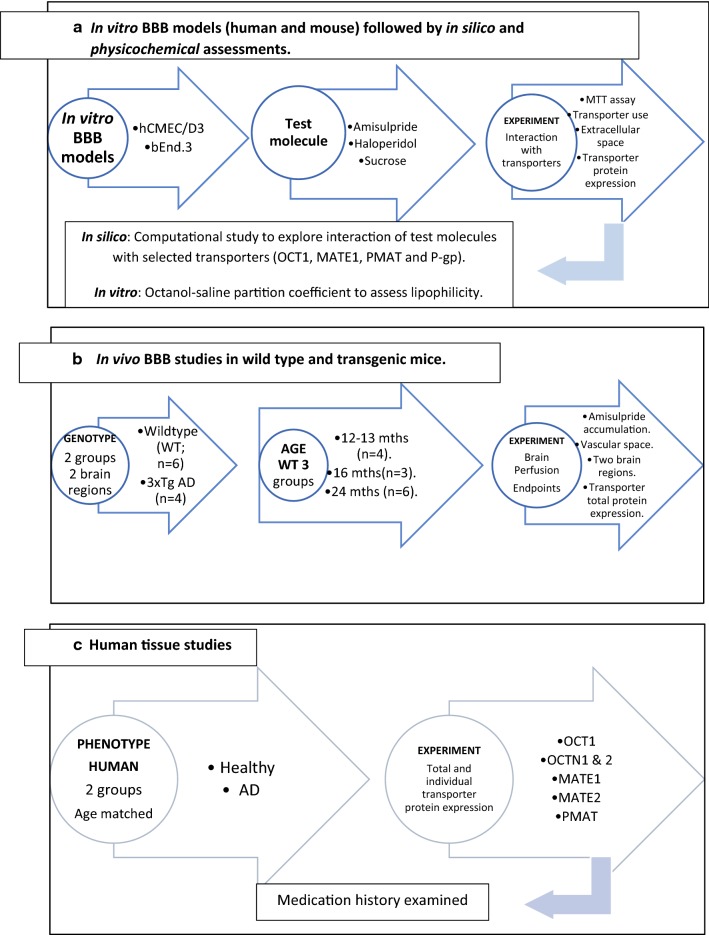
Flow charts to provide an overview of the experimental design for the in silico, in vitro and in vivo approaches. Experiments from the three approaches were performed in parallel

Overall BBB dysfunction in the AD process and its potential impact on drug delivery in particular on antipsychotic medication will be explored (Fig. 1). The results can also be used to inform further studies [27]. Abstracts of this work have been presented [28, 29].

## Materials and methods

### Materials

[O-methyl-^3^H]amisulpride (MW374.8; specific activity 77 Ci/mmol; 97% radiochemical purity) was tritiated (TRQ41291 Quotient, UK). [^3^H(G)]haloperidol (MW375.9; specific activity, 20 Ci/mmol; 99% radiochemical purity: cat# ART1729) was purchased from American Radiolabelled Chemicals Inc, St. Louis, Missouri, USA. [^14^C(U)]sucrose (MW359.48; specific activity 536 mCi/mmol; 99% radiochemical purity: cat# MC266) was purchased from Moravek Biochemicals, USA. Amisulpride (MW369.5, > 98% purity) was purchased from Cayman Chemicals, UK (cat#71675-85-9). Haloperidol (MW375.9; > 98% purity) was purchased from Sigma-Aldrich, Dorset, UK (cat#H1512). Anti-SLC22A1 antibody (Cat#ab55916; RRID:AB_882579), Anti-SLC22A2 antibody (Cat#ab170871: RRID:AB_2751021, Anti-SLC22A3 antibody (Cat#ab183071; RRID:AB_2751016), Anti-SLC22A4 antibody (Cat#ab200641; RRID:AB_2751017), Anti-SLC22A5 antibody (Cat#ab180757; RRID:AB_2751018), Anti-SLC47A1 antibody (Cat#ab104016: RRID:AB_10711136), Anti-SLC47A2 (Cat#ab174344: RRID:AB_2751019), SLC29A4 antibody (Cat#ab56554: RRID:AB_2190909), Goat Anti-Rabbit IgG H&L (cat#ab6721: RRID:AB_955447) and Rabbit anti-mouse HRP (Cat#ab6728: RRID:AB_955440) were purchased from Abcam, UK. Anti-SLC47A1 (Cat#ab174344: RRID:AB_2751019) was purchased from Alomone Laboratories, Israel. Anti-SLC29A4 (Cat#: bs-4176R: RRID:AB_11108960) was purchased from Bioss antibodies, USA. Goat anti-rabbit (IgG)-HRP was purchased from (Cell Signalling. Cat#7074S:AB_2099233). Transferrin receptor monoclonal antibody was purchased from (Thermo Fisher Scientific, CAT# 13-6800 RRID: AB_2533029). Table 1 details the dilutions used. All consumables were purchased within the years 2012 to 2018 except the transferrin receptor antibody which was purchased in 2019.

**Table 1:** Primary and secondary antibodies used for protein expression studies

Protein	Primary antibody	Secondary antibody Western blot (WB)
OCT-1 (SLC22A1)	Rabbit polyclonal anti-human and mouse (Abcam, Cat#ab55916; RRID:AB_882579), WB dilution—1:250	Goat anti-rabbit HRP (Abcam, cat#ab6721: RRID:AB_955447) dilution—1:1000
OCT-2 (SLC22A2)	Rabbit monoclonal to human and mouse (Abcam, Cat#ab170871: RRID:AB_2751021), WB dilution—1:2000	Goat anti-rabbit HRP (Abcam, cat#ab6721: RRID:AB_955447) dilution—1:2000
OCT-3 (SLC22A3)	Rabbit polyclonal to human and mouse (Abcam, Cat#ab183071; RRID:AB_2751016), WB dilution—1:600	Goat anti-rabbit HRP (Abcam, cat#ab6721: RRID:AB_955447) dilution—1:2000
OCTN1 (SLC22A4)	Rabbit polyclonal to human and mouse (Abcam, Cat#ab200641; RRID:AB_2751017), WB dilution—1:1000	Goat anti-rabbit (IgG)-HRP (Cell Signalling. Cat#7074S:AB_2099233) dilution—1:1000
OCTN2 (SLC22A5)	Rabbit polyclonal to human and mouse (Abcam, Cat#ab180757; RRID:AB_2751018), WB dilution—1:1000	Goat anti-rabbit (IgG)-HRP (Cell Signalling. Cat#7074S: AB_2099233) dilution—1:1000
MATE1 (SLC47A1)	Rabbit polyclonal to human from Abcam (Cat#ab104016: RRID AB_10711136), WB dilution—1:500	Goat anti-rabbit HRP (Abcam, Cat#ab6721: RRID:AB_955447) dilution—1:2000
MATE1 (SLC47A1)	Rabbit polyclonal to mouse from Alomone labs (Cat#ANT-131: RRID:AB_2751020), WB dilution—1:800	Goat anti-rabbit HRP (Abcam, Cat#ab6721: RRID:AB_955447) dilution—1:2000
MATE2 (SLC47A2)	Rabbit polyclonal to human and mouse (Abcam, Cat#ab174344: RRID:AB_2751019), WB dilution—1:500	Goat anti-rabbit HRP (Abcam, Cat#ab6721: RRID:AB_955447) dilution—1:2000
PMAT (SLC29A4)	Mouse monoclonal to human and rat (Abcam, Cat#ab56554: RRID:AB_2190909), WB dilution—1:500	Rabbit anti-mouse HRP (Abcam, Cat#ab6728: RRID:AB_955440) dilution 1:2000
PMAT (SLC29A4)	Rabbit polyclonal to human, mouse and rat (Bioss Antibodies; Cat#:bs-4176R: RRID:AB_11108960), WB Dilution—1:800 for hCMEC/D3—1:650 for b.End3, 1:600 mouse capillaries	Goat anti-rabbit (IgG)-HRP (Cell Signalling. Cat#7074S: RRID:AB_2099233) dilution—1:1000
TfR	Transferrin receptor monoclonal antibody (Thermo Fisher Scientific, CAT# 13-6800: RRID: AB_2533029), WB Dilution—1:1000	Rabbit polyclonal Secondary to Mouse IgG—HRP (Abcam, CAT# ab6728 RRID: 955_440) WB dilution—1:1000
GAPDH	Rabbit polyclonal to GAPDH (Abcam, Cat#ab9485: RRID:AB_307275), WB dilution 1: 2500 or 1:10,000	

The predicted antibodies with their predicted molecular weight (MW) WB were all made up in PBS-T with 5% BSA. Actual band sizes may differ due to post translational modifications or cleavages. (Validation data is available from the Abcam website (https://www.abcam.com/nav/primary-antibodies), cell signalling technology website (https://www.cellsignal.co.uk) and Alonome labs website (https://www.alomone.com). Accessed 19.10.18. Validation data is available from the BIOSS USA antibodies website (http://www.biossusaantibodies.com). Accessed 4.12.18. Verification data is available from ThermoFisherScientific (https://www.thermofisher.com). Accessed 20.11.2019

### In vitro model of the BBB

#### Cell culture

The hCMEC/D3 (human) and bEnd.3 (mouse) are well-established models of the BBB [21, 30–32]. They are not listed on the misidentified cell line register (version 9 released 14th October 2018). Both lines require different mediums to grow to confluence and their BBB phenotype has been confirmed using Western blots, confocal and transmission electron microscopy [32] (“Western Blot procedure” section). Functional expression of several transporters has been demonstrated by our group [32–34].

- hCMEC/D3 cells (passages 27–35) were provided under a MTA and maintained in Clonetics^®^ endothelial cell growth medium-2 MV Bullet Kit (Cat# CC-3162 Lonza, UK) containing the endothelial basal medium, the SingleQuotsTM growth factor kit, foetal bovine serum (FBS), penicillin–streptomycin and HEPES (Sigma-Aldrich, UK) [32, 34, 35].
- bEnd.3 cells (passages 17–25), isolated from the SV129 strain of mice and transformed with the Polyoma virus middle T-antigen, were purchased from ATCC^®^ and underwent authentication tests during the accessioning process (CRL-2299: RRID:CVCL0170) [36]. They were grown in T-75 flasks (Fisher Scientific, cat# 15350591) using high glucose Dulbeccos Modified Eagles Medium (Sigma-Aldrich, UK, cat# D6429) supplemented with 10% FBS (vol/vol) and 1% penicillin–streptomycin (vol/vol: Fisher Scientific cat#10003927).

Both lines were maintained at 37 °C/5% CO_2_ in an incubator with saturated humidity. Medium was changed every 2–3 days. Cells were split when they reached 80–90% confluency and were seeded onto 96-well plates (ThermoScientific, UK) at 20,000 cells/cm^2^ for b.End3 cells and 25,000 cells/cm^2^ for hCMEC/D3 cells. The hCMEC/D3 96-well plates were pre-coated with 0.1 mg/ml rat tail collagen type1 (Gibco cat#A1048301).

Both lines were confluent in 4–5 days and were left for another 4 days to allow for further differentiation before experimentation. The medium was changed every 2–3 days.

#### Drug accumulation assay

Accumulation assays were performed on confluent cell monolayers grown in the centre 60 wells of 96-well plates. Each passage was regarded as one ‘n’. No sample calculation was performed. The accumulation buffer (pH7.4) composition was 135 mM NaCl, 10 mM HEPES, 5.4 mM KCl, 1.5 mM CaCl_2_, 1.2 mM MgCl_2_, and 1.1 mM d-glucose and water. It also contained 3.7–7.7 nM [*O*methyl-^3^H]amisulpride and 9.4 µM [^14^C(U)]sucrose or 10 nM [^3^H]haloperidol and 3.8 μM [^14^C]sucrose. [^14^C]sucrose is a similar size to the test molecules and was used as an inert marker of extracellular space and membrane integrity. After the exposure period, buffer was aspirated and the wells washed with ice-cold PBS^+^ (Sigma-Aldrich, UK) to remove drug that was not taken up by cells and to stop further transport. 1% Triton X-100 (Sigma-Aldrich) was added and the plate was incubated for an hour at 37 °C to lyse the cells and to release accumulated [^3^H]drug. 100 µl from each of the wells was transferred to a vial and scintillation fluid (4 ml) added (Optiphase Hisafe 2, PerkinElmer, UK). Radioactivity was measured using a Packard Tri-Carb 2900TR liquid scintillation counter (PerkinElmer, UK) and corrected for background. The remaining 100 µl in each well were used to perform a bicinchoninic acid (BCA) protein assay. A range of 2–30 μl mg^−1^ of protein was acceptable. All data for [^3^H]amisulpride or [^3^H]haloperidol were expressed as a volume of distribution (V_d_) after correction for [^14^C]sucrose. The V_d_ was calculated from the sum of accumulated radioactivity (a sum of efflux and influx of the molecule) (disintegrations per minute (dpm)/mg protein) over the ratio of dpm/μl of accumulation buffer. Outliers were identified by examining the [^14^C]sucrose values. [^14^C]sucrose values are presented in the figures and tables.

#### Transporter inhibition assay

Transporter interaction was examined by incubating [^3^H]amisulpride and [^14^C]sucrose with potential inhibitors. These included unlabelled amisulpride and inhibitors/substrates of OCT, OCTN, MATE, PMAT, P-gp, BCRP, and MRP at established concentrations (Additional file 1: Table S1). The MATE1 inhibitor, famotidine, was also utilized at a higher concentration of 2 μM. ATP depletion (Promega Enliten assay) was carried out by pre-incubation with the glycolysis inhibitor, 10 mM 2-deoxy-d-glucose, for an hour before incubating the cells with [^3^H]amisulpride and [^14^C]sucrose. This assay had previously been shown to inhibit drug efflux by our group [32, 34].

#### Cytotoxicity assay

Cytotoxicity of amisulpride (0.1–20 μM) and eflornithine (250–500 μM) on both cell lines was assessed using 3-(4,5-dimethylthiazol-2-yl)-2,5-diphenyltetrazolium bromide (MTT) assay [32]. The results were expressed as a percentage of cell viability. This assay was also utilized on hCMEC/D3 cells (passage 32 and/or 33) for 2 h to assess toxicity of 1 μM famotidine, 2 μM famotidine, 2 μM lopinavir, 20 μM ergothioneine, 5 μM l-carnitine, 3 μM nifekalant hydrochloride and 1.5 μM [^14^C]sucrose. We have already published MTT assay results for eflornithine (250 μM) in hCMEC/D3 cells and haloperidol (40 μM), dexamethasone (200 μM), pentamidine (10 μM), ko143 (1 μM), MK571 (10 μM), amantadine (500 μM), corticosterone (50 μM), pheophorbide A (1 μM) and prazosin (100 μM) in both cell lines [32, 34]. No significant effect was observed except with prazosin on bEnd.3 cells.

### Lipophilicity

Lipophilicity is a standard physicochemical measure which is very important in terms of understanding drug distribution across membranes including the BBB and can be expressed in the form of an octanol-saline partition coefficient. The higher the lipophilicity the greater the ability of the molecule to cross the plasma membrane by passive diffusion. An octanol-saline partition coefficient for [^3^H]amisulpride and [^3^H]haloperidol was determined [37].

### In silico computational study

Using in silico molecular docking, we tested the molecular level interactions of amisulpride, haloperidol and prazosin (OCT1 and OCT3 substrate) with the transporters OCT1, PMAT and MATE1. Due to the unavailability of the crystal structures of these transporters, molecular models of OCT1, PMAT and MATE1 were developed using homology modelling with Swiss-model webserver using PDB codes 4PYP, 5Y50 and 4ZOW, respectively, as templates (Additional file 1). A further molecular docking study was performed to explore the interaction of amisulpride, dexamethasone (P-gp substrate) and colchicine (P-gp substrate) in the binding site of the multidrug transporter ABCB1 (P-glycoprotein), and the pbd code 6FN1 [38] was used as the template. Molecular docking was performed using Dock Ligands (CDOCKER) protocol from Discovery studio version 4.0. CDOCKER is an implementation of a CHARMm based docking tool where each orientation is subjected to simulated annealing molecular dynamics. The binding sites were chosen after comparing the docking results of the ligands in all possible binding cavities within the transporters.

### Animal model of AD

All experiments were performed in accordance with the Animal Scientific Procedures Act (1986) and Amendment Regulations 2012 and with consideration to the ARRIVE guidelines. The study was approved by the King’s College London Animal Welfare and Ethical Review Body and performed under license: 70/7755. A total of 40 mice were used for this study. Mice were housed at King’s College London. C57BL6/129 mice (wild-type) and the triple transgenic AD model (3×TgAD) of C57 mice which harbour the human APP-Swedish mutation (KM670/671NL), tau mutation (P301L), and presenilin-1 mutation (M146V) were utilized at 12–13 (mid-age), 16 (old) or 24 (elderly) months old. The 3×TgAD is an established AD model displaying the temporal and spatial progression and mirroring the neuropathological development seen in AD [39]. These mice were bred and genotyped at King’s College London and were a gift. They had originally been supplied by the Jackson Laboratory (RRID:IMSR_JAX:008880). No sample size calculation was performed and the study was not pre-registered. Previous experience suggested that even with the limited number of animals available there would still be sufficient power (95%) to detect significant differences (p < 0.05) [40]. Welfare was assessed daily by animal technologists. Animals were identified by earmarks and housed together by age and genotype in guideline compliant cages. All animals were maintained under standard temperature/lighting conditions and given food and water ad libitum. The experiment on each animal had to be performed within set time frames to allow the three age groups to be achieved. Experiments were performed between 9am and 5 pm. The experimenter was not blinded.

The median life span of 3×TgAD mice has been reported to be 673 days (22 months) which is shorter than the 907 day (30 months) lifespan of C57BL/6J [41]. Adult male BALB/c mice (inbred strain) were purchased from Harlan UK Limited (Oxon, UK). All mice were anaesthetised (2 mg/kg i.p. medetomidine hydrochloride and 150 mg/kg i.p. ketamine) and heparinized (100 U i.p.) in a procedure room separate to the laboratory where the brain perfusion was performed. Advice was sought from the named veterinary surgeon regarding the anaesthetic.

#### In situ brain perfusion

To assess differences in [^3^H]amisulpride and [^14^C]sucrose transport into the brain in ageing and in AD, wildtype (C57BL6/129 n = 17) and transgenic AD (3×Tg n = 6) mice were used. Perfusion (10 min; 5 ml/min) with a warmed (37 °C) and oxygenated (95% O_2_; 5% CO_2_) artificial plasma was via a cannula in the left ventricle of the heart as previously described [42]. The artificial plasma consisted of a modified Krebs–Henseleit mammalian Ringer solution with the following constituents: 117 mM NaCl, 4.7 mM KCl, 2.5 mM CaCl_2_, 1.2 mM MgSO_4_, 24.8 mM NaHCO_3_, 1.2 mM KH_2_PO_4_, 10 mM glucose, and 1 g/l bovine serum albumin. With the start of perfusion the right atrium of the heart was sectioned to prevent the recirculation of the artificial plasma. At the end of perfusion the animal was decapitated and the brain removed. To determine the brain concentration of [^3^H]amisulpride, frontal and occipital cortex samples were taken and weighed using a Leica S4E microscope (Purchased 2005). Brain samples were then solubilized with 0.5 ml of Solvable (PerkinElmer, USA) and liquid scintillation fluid (3.5 ml; Lumasafe; PerkinElmer) added. Radioactivity in the samples were determined using the Tri-Carb2900TR scintillation counter (Purchased 2007).

#### Expression of results

Radioactive concentrations in the brain samples (dpm/g) were expressed as a percentage of that in the artificial plasma (dpm/ml) and termed R_TISSUE_ (millilitres/100 g). All R_TISSUE_ values for [^3^H]amisulpride were corrected for vascular/extracellular space by subtracting the [^14^C]sucrose R_TISSUE_ value. Examination of the [^14^C]sucrose values (i.e. vascular space) and comparison to previously published values determines if the result is an outlier. This is acceptable for the WT animals, however, it is noted that [^14^C]sucrose (vascular space) may be affected in AD. No outliers were detected in this study.

### hCMEC/D3 and b.End3 monolayers isolation

In order to perform Western blots, cell lines were grown to confluence in T-75 flasks (Thermo Scientific, UK) and left for 3–4 days, as previously described. The flask was then transferred to ice and the medium removed, before the cells were washed twice using ice-cold PBS+. Then, 1 ml of ice-cold Radio-Immunoprecipitation Assay (RIPA) buffer (Sigma-Aldrich, Dorset, UK) with added protease inhibitors (10% v/v) (Thermo Scientific, Loughborough, UK) was added to the flask to lyse the cells. A plastic cell scraper (Greiner Bio-One Ltd, Gloucestershire, UK) was used to scrape the cells off the bottom of the flask and the cell lysate was transferred to a pre-cooled 1.5 ml Eppendorf tube which was left on ice for 20 min. The tubes were then centrifuged at 10,000 rpm for 10 min at 4 °C using a Thermo Electron Corporation Heraeus Fresco17 bench-top micro-centrifuge. After centrifugation, the supernatant was transferred to another pre-cooled 1.5 ml Eppendorf and the pellet discarded. The resulting supernatant was taken for Western blot analysis.

### Mouse brain capillary isolation

Brain capillaries from old-age (16 months) wild-type (3 females) and 3×TgAD (3 males, 1 female) mice were used to explore MATE1 expression. Brain capillaries were also isolated from elderly (24 month) age-matched wild-type C57BL6/129 mice (3 males, 2 females) and 3×TgAD mice (3 males, 2 females) for all other transporter studies. The left ventricle of the heart was cannulated and perfused (5 ml/min) with an oxygenated artificial plasma (modified Krebs–Henseleit mammalian Ringer) for up to 2 min. The right atrium was sectioned before perfusion was started. The mice were then decapitated and the perfused brain removed. The brain was homogenized in physiological buffer (brain weight × 3) and 26% dextran (brain weight × 4). The homogenate was subjected to density gradient centrifugation (5400×*g* for 15 min at 4 °C) to give an endothelial cell-enriched pellet and the supernatant was discarded [42]. 300 µl of ice-cold RIPA: ThermoFisher Scientific cat#89900) buffer with added protease inhibitors was added to the pellet at 4 °C to lyse the tissue and then centrifuged at 8000×*g* for 15 min at 4 °C. The resulting supernatant was taken for Western blot analysis.

### Human tissue

Human tissue was provided with informed consent via the brains for dementia research (BDR) and were anonymized. BDR has ethical approval granted by the national health service (NHS) health research authority (NRES Committee London-City & East, UK: REC reference: 08/H0704/128+5. IRAS project ID:120436). Tissue was received on the basis that it will be handled, stored, used and disposed of within the terms of the Human Tissue Act 2004. Post-mortem brain capillaries from healthy individuals (Braak stage 0–II; 86.8 ± 1.5 years; 2 females, 3 males) and AD cases (Braak stage V–VI; 79.4 ± 3.7 years; 2 females, 3 males) were used to investigate the expression of transporters (Case details—Additional file 1: Table S2). Medication history of the cases was supplied by the Manchester Brain Bank (Additional file 1: Table S3). In this study we identified those drugs prescribed as sedatives, antidepressants and antipsychotics.

### Human brain microvasculature isolation

Brain capillaries from frontal cortex, caudate nucleus, and putamen samples were isolated after homogenising 300 mg tissue and carrying out a dextran-based density-gradient centrifugation to produce a capillary-enriched pellet.

The pellet was further lysed with 500 µl of ice-cold RIPA buffer with added protease inhibitors at 4 °C and then centrifuged at 8000×*g* for 15 min at 4 °C. The resulting supernatant was taken for Western blot analysis to examine transporter expression. The presence of transferrin receptor in the supernatant indicated that the method generated samples containing capillary endothelial cells.

### Western Blot procedure

The supernatant protein concentration was determined using a BCA assay (Albumin standard, ThermoScientific). The supernatants were diluted and boiled for 5 min at 95 °C in 5× Laemmli sample buffer. Cell lines (30 μg except for MATE 1 antibody in Bend.3 cells where 15 μg was utilized and PMAT antibody in hCMEC/D3 and bEnd.3 cells where 20 μg and 10 μg was utilized respectively), mouse samples (15 μg for MATE1, OCTN1 and 2) and (30 μg for MATE2, PMAT and OCT1), human samples (10 μg for OCNT1 and 2) or 15–20 μg (for MATE1, MATE2, PMAT and OCT1) were loaded equally on 4–20% Mini-PROTEAN^®^ TGX™ gels (Bio-Rad) alongside a molecular weight marker (Precision plus protein, Bio-Rad). Samples underwent SDS-PAGE at 160 V for 1 h. Proteins were transferred onto 0.45 μm polyvinylidene fluoride membranes (GE Healthcare, UK) after methanol activation at 100 V for 1 h. Membranes were blocked to reduce nonspecific binding using 5% milk with PBS-TWEEN^®^ tablets (PBS-T) (Calbiochem, USA) at room temperature (RT) for 1 h. Membranes were incubated overnight at 4 °C with primary antibodies in PBS-T (Table 1). Membranes were washed in PBS-T (3 × 10 min) and incubated with the secondary antibody in PBS-T at RT for 1 h. Further washing in PBS-T (3 × 10 min), membranes were then incubated with enhanced chemiluminescent reagent (ThermoScientific) for 30 s at RT.

Quantification of protein expression was determined by calculating the intensity ratio of the band of interest and the band of the loading control (tubulin or GAPDH). Band intensity ratio analysis was conducted using ImageJ software (NIH). Our group have previously published results from Pgp, BCRP, OCT1, OCT2 and OCT3 protein expression studies of bEnd.3 and hCMEC/D3 cells [32, 34].

### Data analysis

Data are expressed as mean ± SEM. The data was analysed by two-way ANOVA with Holm-Sidak post hoc test for the accumulation studies and in situ perfusion studies, one-way ANOVA with Tukey’s post hoc test for MTT assay and Student’s t-test or two-way ANOVA for Western blot data using Sigmaplot version 13 (Systat, USA) or GraphPad Prism 7.03. p < 0.05 were considered as statistically significant. Exact p-values are provided in figure legends/“Results” section.

## Results

### Amisulpride accumulation and saturable transport

**Fig. 2:**
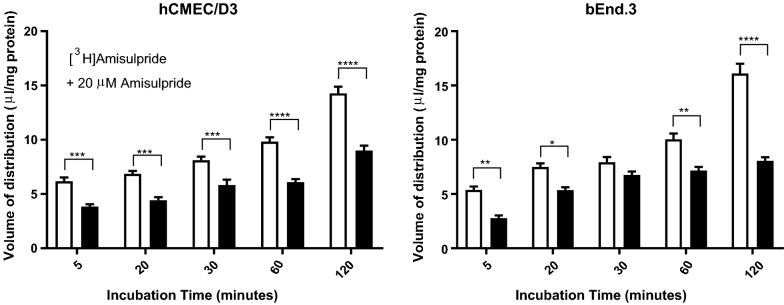
The effect of self-inhibition (20 μM) on the accumulation of [^3^H]amisulpride (6.5 nM) was determined in hCMEC/D3 (**a**) and bEnd.3 (**b**) cell lines. Significant differences compared to control were observed—*p ≤ 0.05, **p ≤ 0.01, ***p ≤ 0.001, ****p ≤ 0.0001. All data have been corrected for [^14^C]sucrose and are expressed as mean ± S.E.M, n = 3 to 7 plates with 6 replicates (wells) per timepoint per plate (5 time-points)

[^3^H]Amisulpride was able to accumulate in both hCMEC/D3 and bEnd3 cell lines to a greater extent than the baseline marker, [^14^C]sucrose (Fig. 2 and Additional file 1: Figure S1). Incubation of hCMEC/D3 and bEnd3 cell lines with 20 µM amisulpride significantly decreased the accumulation of [^3^H]amisulpride (6.5 nM) at 5 min—with a significant decrease of 37% in hCMEC/D3 cells and a decrease of 50% in bEnd.3 cells observed after 2 h (Fig. 2). No significant differences were observed for [^14^C]sucrose between the treatments, except in the bEnd3 cells at 120 min where the presence of 20 μM amisulpride decreased the accumulation of [^14^C]sucrose (Additional file 1: Figure S1). However, all [^14^C]sucrose values were within the expected range for these in vitro models. Studies also revealed that lower concentrations of unlabelled amisulpride (0.1 μM) did not affect accumulation of [^3^H]amisulpride in hCMEC/D3 (n = 5 passages) or bEnd.3 (n = 3 passages) cells at all time points (data not shown).

### ABC transporter involvement

ATP depletion did not affect the accumulation of [^3^H]amisulpride or [^14^C]sucrose in either cell line (Additional file 1: Figure S2). The P-gp substrate, dexamethasone, BCRP substrate, ko143, and inhibitor, pheophorbide A, and MRP family inhibitor, MK571, did not affect the accumulation of [^3^H]amisulpride or [^14^C]sucrose in either cell line (Additional file 1: Figure S3).

### OCT, OCTN, PMAT and MATE involvement

Involvement of OCTs in [^3^H]amisulpride uptake was investigated by incubating hCMEC/D3 and bEnd.3 cells with the, OCT1 and 2 substrate, amantadine, the OCT1 and 3 substrate, prazosin, and the OCT3 substrate, corticosterone (Additional file 1: Figure S4). [^3^H]amisulpride accumulation did not change in the presence of amantadine in hCMEC/D3 cells, but significantly increased by 84% in bEnd.3 cells. In the presence of prazosin, there was a significantly reduced accumulation of [^3^H]amisulpride in hCMEC/D3 cells, but not in bEnd.3 cells. Corticosterone did not affect the accumulation of [^3^H]amisulpride in either cell line. No differences were found for [^14^C]sucrose between the treatments (Additional file 1: Figure S4).

[^3^H]amisulpride accumulation in hCMEC/D3 and b.End3 was unaffected by the presence of ergothioneine (OCTN1) and l-carnitine (OCTN2) respectively (Additional file 1: Figures S5 and S6). [^14^C]Sucrose V_d_ was not significantly different between the treatments, except significant differences were observed between [^14^C]sucrose and l-carnitine at 2 h suggestive that this time point for [^3^H]amisulpride should be ignored.

**Fig. 3:**
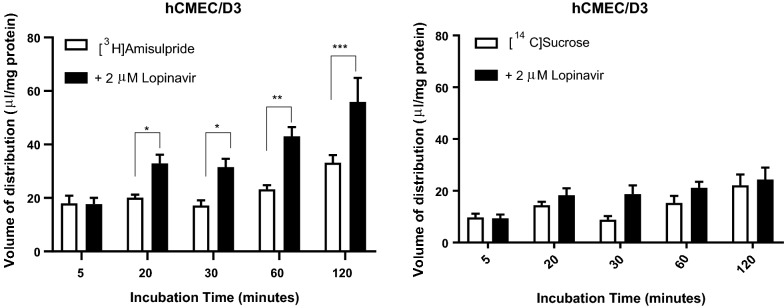
The effect of PMAT inhibition on the accumulation of [^3^H]amisulpride (3.7–7.7 nM) was determined in hCMEC/D3 cell lines. Significant increases were observed compared to control ****p ≤ 0.0001, ***p ≤ 0.001, **p = 0.01 and *p = 0.05. [^3^H]amisulpride data has been corrected for [^14^C]sucrose and are expressed as mean ± S.E.M, n = 5 passages (p30, 2 × p31, p32 and p34 for PMAT) with 6 replicates (wells) per timepoint per plate (5 time-points)

Incubation with the PMAT inhibitor, lopinavir, resulted in a significant increase in the V_d_ of [^3^H]amisulpride by 63.9% at 20 min, 83.2% at 30 min, 85.1% at 60 min and by 68.6% at 120 min (Fig. 3). The V_d_ of [^14^C]sucrose was not significantly different between control and test groups (Fig. 3). No affect was observed with this inhibitor in the b.End3 cells (Additional file 1: Figure S7).

**Fig. 4:**
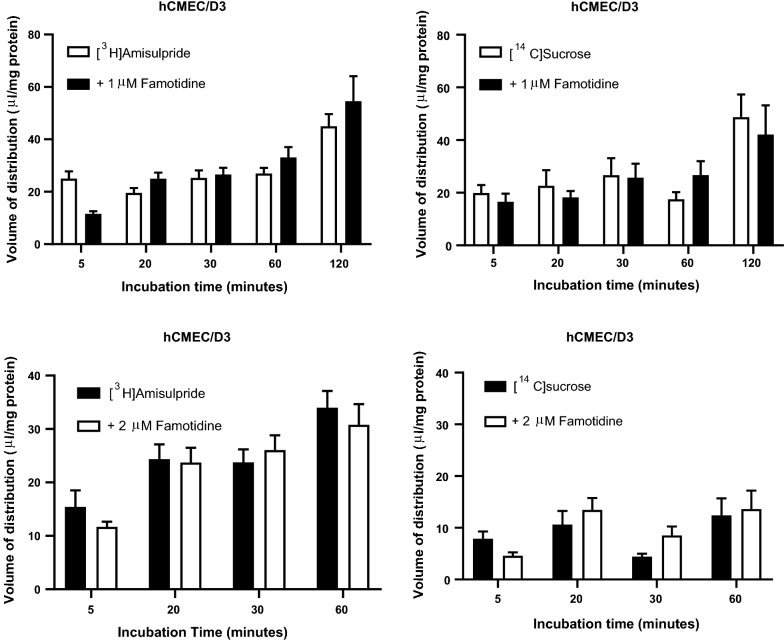
The effect of MATE1 inhibition on the accumulation of [^3^H]amisulpride (3.7–7.7 nM) was determined in hCMEC/D3 cell lines. Significant increases were observed compared to control ****p ≤ 0.0001, ***p ≤ 0.001, **p = 0.01 and *p = 0.05. [^3^H]amisulpride data has been corrected for [^14^C]sucrose and are expressed as mean ± S.E.M, n = 4 passages (p30 × 2, p31 and p34 for MATE1 inhibitor falmotidine at 1 μM and n = 3 passages for MATE1 inhibitor falmotidine at 2 μM) with 6 replicates (wells) per timepoint per plate (5 time-points)

Incubation with the MATE1 inhibitor, famotidine (1 μM) resulted in no significant effect on the V_d_ of [^3^H]amisulpride in hCMEC/D3 cells (Fig. 4). The V_d_ of [^14^C]sucrose was not significantly different between control and test groups with (1 μM) famotidine during the standard 2 h incubation period. An assessment of the effect of 2 μM famotidine on [^14^C]sucrose alone revealed a loss of hCMEC/D3 integrity at 2 h. Further assessment of famotidine (2 μM) did not affect either [^3^H]amisulpride or [^14^C]sucrose accumulation in hCMEC/D3 cells over a 1 h period (Fig. 4). No affect was observed with famotidine (1 μM) in b.End3 with either [^3^H]amisulpride or [^14^C]sucrose (Additional file 1: Figure S7).

Incubation with the MATE2 inhibitor, nifekalant, resulted in no significant affect on the V_d_ of [^3^H]amisulpride in hCMEC/D3 cells (Additional file 1: Figure S8). The V_d_ of [^14^C]sucrose was not significantly different between control and test groups up to 120 min with nifekalant suggesting loss of membrane integrity at this time point. No affect was observed with this inhibitor in b.End3 with either [^3^H]amisulpride or [^14^C]sucrose (Additional file 1: Figure S7).

### Interaction with the positively charged anti-psychotic drug, haloperidol

The effect of the cationic drug, haloperidol (40 μM), on radiolabelled amisulpride accumulation was also investigated. Incubation of unlabelled haloperidol with [^3^H]amisulpride did not yield any significant effects in either cell line (Additional file 1: Figure S9). No significant differences were found for [^14^C]sucrose between the treatments (Additional file 1: Figure S9).

### Characteristics of haloperidol accumulation in hCMEC/D3 and b.End3 cell lines

hCMEC/D3 and b.End3 cells were incubated with unlabelled haloperidol (40 μM) along with [^3^H]haloperidol (10 nM). Incubation with unlabeled haloperidol significantly decreased the accumulation of radiolabelled haloperidol by approximately 93% in hCMEC/D3 cell line and by 94% in bEnd.3 cell line at all times (***p < 0.001) (Additional file 1: Figure S10). No significant differences were found for [^14^C]sucrose between the treatments (Additional file 1: Figure S10).

ATP was depleted from both cell lines to determine the role of ABC transporters in the efflux of haloperidol. ATP depletion did not affect the accumulation of haloperidol in either cell line (Additional file 1: Figure S11). No significant differences were observed for [^14^C]sucrose between the treatments (Additional file 1: Figure S11). The hypothesis that haloperidol uptake is by OCT transporters was investigated by incubating the cells with OCT1 and 2 substrate amantadine (500 μM) and OCT1 and 3 substrate prazosin (100 μM). [^3^H]haloperidol accumulation significantly decreased in the presence of amantadine in both cell lines compared to control—by 89% in hCMEC/D3 cells and by 82% in bEnd.3 cells and in the presence of prazosin—by 85% in hCMEC/D3 cells and by 82% in bEnd.3 cells (***p < 0.001) (Additional file 1: Figure S12). No significant differences were found for [^14^C]sucrose between the treatments (Additional file 1: Figure S12).

The effects of other cationic drugs—unlabeled pentamidine (100 μM), unlabeled efornithine (250 μM) and unlabelled amisulpride (20 μM) on radiolabelled haloperidol accumulation in hCMEC/D3 was investigated. Unlabelled pentamidine significantly reduced the accumulation of radiolabeled haloperidol in the cell lines after 2 h—by 31% in hCMEC/D3 cells (***p < 0.001, **p < 0.01, and *p < 0.05). Unlabelled eflornithine significantly decreased the accumulation of radiolabelled haloperidol by 11% in hCMEC/D3 cells (***p < 0.001). Unlabelled amisulpride (20 μM) significantly decreased the accumulation of radiolabelled haloperidol in hCMEC/D3 cells—by 27% after 2 h (***p < 0.001) (Additional file 1: Figure S13). No significant differences were found for [^14^C]sucrose between the treatments (Additional file 1: Figure S13).

### Cytotoxicity

No cytotoxic effects of amisulpride (0.1–20 μM), 1 μM famotidine, 2 μM famotidine, 2 μM lopinavir, 20 μM ergothioneine, 5 μM l-carnitine, 3 μM nifekalant hydrochloride and 1.5 μM [^14^C]sucrose (Additional file 1: Figure S14A and B) and eflornithine (250–500 μM) were detected using the MTT assay (data not shown). A marker molecule ([^14^C]sucrose) of extracellular/vascular space was included in all [^3^H]amisulpride accumulation experiments and ensured that any measured effect on [^3^H]amisulpride values could be interpreted correctly and was not simply due to loss of membrane integrity caused by the cytotoxic nature of the drugs/inhibitors utilized.

### Lipophilicity

The octanol-saline partition coefficient for [^3^H]amisulpride was determined to be 0.0422 ± 0.0045 and for [^3^H]haloperidol was determined to be 0.6678 ± 0.1278.

### Molecular docking studies with the SLC transporters—OCT1, MATE1 and PMAT

**Fig. 5:**
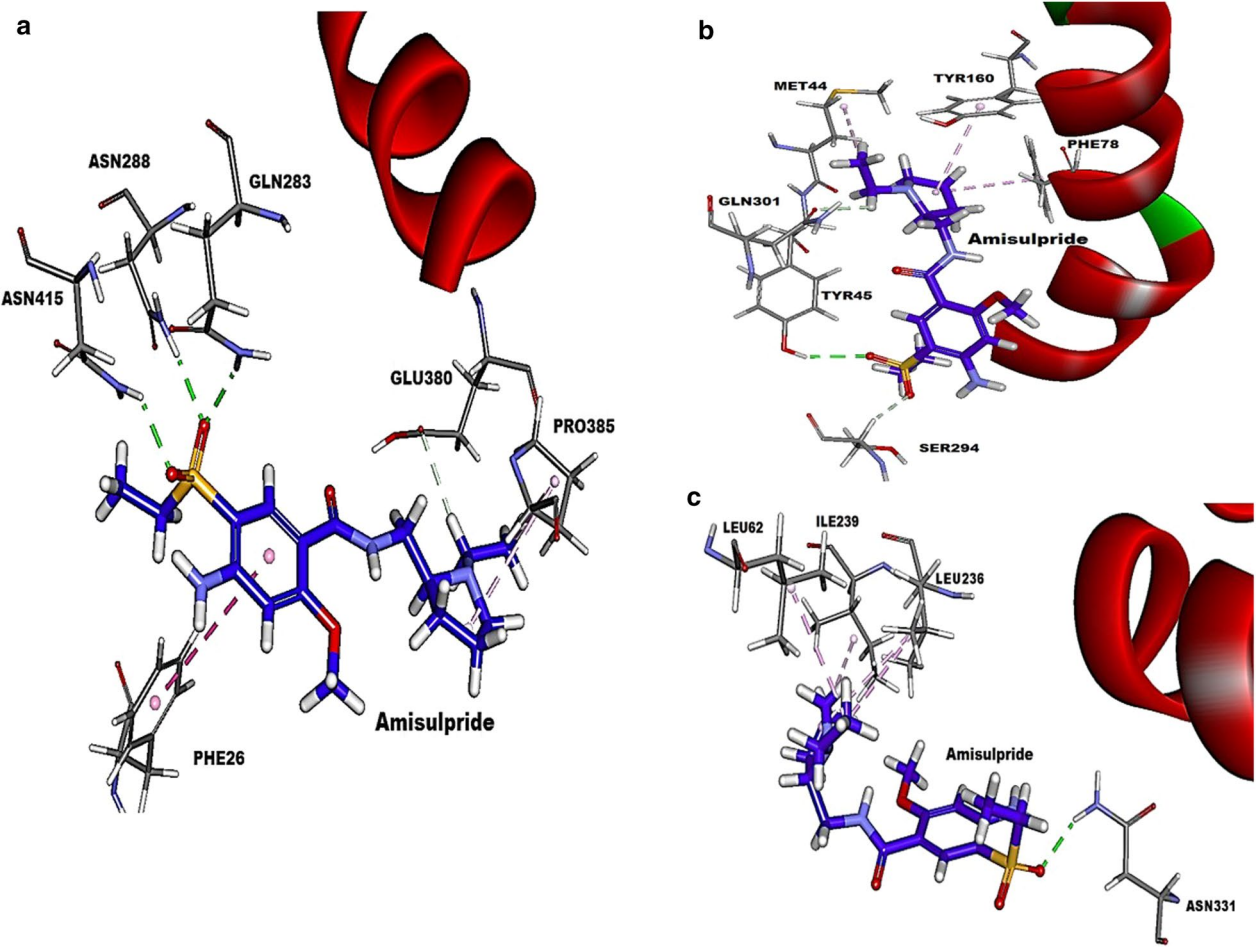
Molecular-level interactions of amisulpride within the binding site of OCT1 (**a**), MATE1 (**b**) and PMAT (**c**). Amisulpride is represented in stick-representation and amino acid residues in line-representations. Hydrogen bonds are represented in green dotted lines, and hydrophobic interactions are represented in pink dotted lines

Amisulpride showed molecular interactions inside the binding site of OCT1 in the form of hydrogen bonds with amino acids Gln283, Asn288, Glu380 and Asn415 as well as hydrophobic interactions with amino acid residues Phe26 and Pro385 (Fig. 5a), while haloperidol showed hydrogen bonds with amino acids Gln 283, Gly384, Trp388 and hydrophobic interactions with Gly384 and Pro385 while prazosin interacted with Asn288, Thr321, Ser324, Glu380, Asn411 through hydrogen bonds and hydrophobic interactions with Leu325, Val328, Gly384 and Pro385 (Additional file 1: Figure S15A). The best pose of amisulpride interacted with the binding pocket of OCT1 with a free energy of binding of − 14.28 kcal/mol while the free energy of binding for haloperidol and prazosin were − 29.97 kcal/mol and − 27.57 kcal/mol.

Amisulpride showed a similar level of interaction with MATE1 transporter with a free energy of binding of − 14.32 kcal/mol. It fit snugly within the binding pocket (Fig. 5b) and formed hydrogen bonds with amino acids Tyr45, Ser294 and Gln301 as well as hydrophobic interactions with amino acid residues Met44, Phe78 and Tyr160. The hydrophobic interactions appeared to play an important role in its interaction with MATE1 compared to its interaction with OCT1. The interaction of amisulpride was relatively weaker with PMAT compared to both OCT1 and MATE1 with free energy of binding − 11.4 kcal/mol. It formed a single hydrogen bond with Asn331 and interacted with hydrophobic interactions with amino acid residues Leu62, Leu236 and Ile239 through hydrophobic interactions (Fig. 5c).

A similar molecular docking study suggested haloperidol is a better substrate of both MATE1 and PMAT compared to amisulpride as it showed binding affinity of binding − 22.27 kcal/mol and − 21.72 kcal/mol for MATE1 and PMAT, respectively, which are notably higher than amisulpride (Fig. 5 and Additional file 1: Figure S16). It showed good interaction with MATE1 with hydrogen bonds with Tyr45 and Ser74 and hydrophobic interaction with Phe82 and Ala67 (Additional file 1: Figure S16A). However, the interaction of haloperidol with PMAT was limited to single hydrogen bond with Asp34 and hydrophobic interaction with Leu62 (Additional file 1: Figure S16B).

### Molecular docking studies with the ABC transporter—P-gp

The molecular docking study revealed that amisulpride was not a substrate for P-gp with a free energy binding of − 1.81 kcal/mol and the molecule was not able to interact favourably with the binding pocket of P-gp. P-gp substrates, dexamethasone and colchicine, showed notably superior interaction with P-gp with free energy of binding values of − 31.83 kcal/mol and − 16.07 kcal/mol. Both dexamethasone and colchicine interacted with the binding pocket employing hydrogen bonds and hydrophobic interactions (Additional file 1: Figure S17 and Table S4).

### CNS amisulpride delivery in vivo

**Fig. 6:**
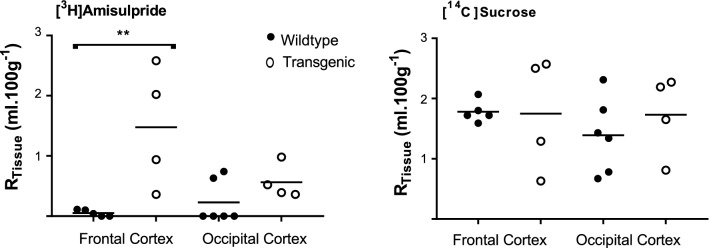
The uptake of [^3^H]amisulpride was determined in wildtype and 3×transgenic AD mice. Significant differences were observed for [^3^H]amisulpride between wildtype (n = 5 frontal cortex and n = 6 occipital cortex) and transgenic mice (n = 4 each region)—**p < 0.005. [^3^H]Amisulpride data have been corrected for [^14^C]sucrose. [^14^C]Sucrose uptake is shown. No differences in paracellular permeability and membrane integrity were observed. All data are expressed as mean ± S.E.M, n = 4–6 mice, 2 years old. Perfusion time was 10 min. 6 C57BL6/129 mice (3 males and 3 females: weight 37.0 ± 1.8 g) and 4 transgenic (2 males, 2 females: weight 29.1 ± 1.0 g) were used. Also see Additional file 1: Table S5

The R_TISSUE_ values for [^3^H]amisulpride did not differ from the R_TISSUE_ values for [^14^C]sucrose in wildtype mice in all the age groups tested (Additional file 1: Table S5). There was also no effect of ageing on the brain distribution of [^3^H]amisulpride or [^14^C]sucrose. No differences were observed in the [^14^C]sucrose R_TISSUE_ values in the frontal and occipital cortex between the wildtype and transgenic mice (Fig. 6). However, in transgenic mice the R_TISSUE_ value for [^3^H]amisulpride was significantly higher than [^14^C]sucrose in the frontal cortex, but not the occipital cortex. Importantly, in transgenic mice the sucrose-corrected R_TISSUE_ value for [^3^H]amisulpride was significantly higher than that of wildtype mice in the frontal cortex, but not the occipital cortex (Fig. 6).

### Endothelial transporter expression

#### Cell lines

OCTN1, OCTN2, MATE1 and MATE2 expression was confirmed in hCMEC/D3 (passages 28 and 33) and bEnd.3 (passages 18, 19 and 23) cells (Additional file 1: Figures S18 and S19A). PMAT was expressed in hCMEC/D3 cells (passages 28, 31 and 32) and bEnd.3 (passages 17, 18, 20 and 24) (Additional file 1: Figure S19B and C).

#### Wildtype and transgenic AD mice

The total protein concentration measured in wildtype mice (188.2 ± 12.8 μg/100 μl) was not significantly different to that measured in 3×Tg AD mice (195.6 ± 13.3 μg/100 μl) brains, but this may be attributed to the fact that it was not possible to assess regional differences in these small samples. Although slight variability was observed, there was no significant differences in individual transporter (P-gp, OCT1, OCT2, OCT3, OCTN1, OCTN2, MATE1, MATE2 and PMAT) expression between the wildtype and 3×TgAD mice (Additional file 1: Figures S20 and S21). Note mice were 24 months old except for MATE1 and PMAT studies where the mice were 16 and 12 months old. Data not shown for P-gp, OCT1, OCT2, OCT3 and PMAT. The expression of all these BBB transporters suggests that a capillary enriched sample had been assessed.

#### Human brain

Human brain capillaries were isolated from the frontal cortex (for comparison with in situ perfusion experiments), caudate nucleus and the putamen (forming the striatum where high D2 and D3 receptor occupancy is observed in individuals with AD with amisulpride usage) of healthy controls and age-matched AD affected individuals (Additional file 1: Tables S2 and S3). The total protein concentration in the capillaries was found to be significantly lower in the caudate nucleus (by 37.4%) and putamen (by 32.5%), but not the frontal cortex samples, from individuals with AD compared to healthy controls (Additional file 1: Table S6).

**Fig. 7:**
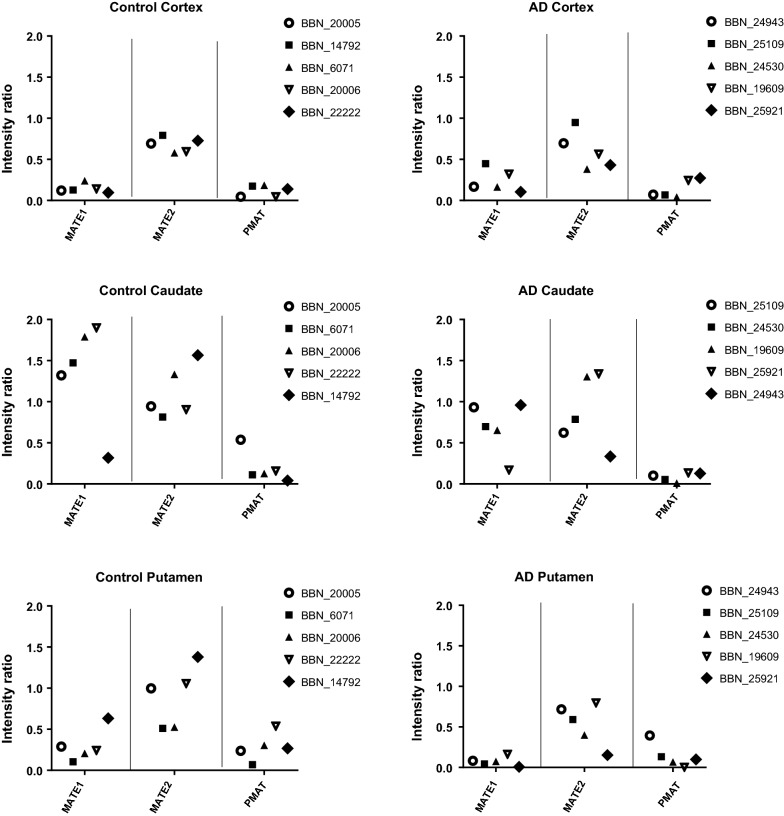
Individual values have been plotted for the transporter expression in the capillaries of frontal cortex, caudate nucleus and caudate putamen samples from healthy and AD affected individuals. The numbers in the key indicate the MRC ID designated to each sample. Details of the samples can be found in Additional file 1: Tables S2 and S6

Individual transporter expression in each brain region between AD and healthy cases is comparable as the same amount of protein has been loaded into each well. Note this amount was dependent on the antibody utilized so was variable. Transporter expression in the frontal cortex was less variable between healthy and AD cases than in the other regions studied with no significant differences in transporter expression being observed (Additional file 1: Figures S22–S28; Fig. 7). Expression of OCT1 did not change between control and individuals with AD in all the regions tested (Additional file 1: Figures S22, S23). PMAT and MATE1 expression was significantly lower in individuals with AD in the caudate nucleus (56.2%; p < 0.05) and putamen (74.8%; p < 0.05) samples, respectively, compared to control (Fig. 7; Student’s t-test). No other significant differences were observed, however, further cases are required to explore this more fully. Please note it is also likely that transporter expression in caudate nucleus and putamen AD samples is even lower than the heathy controls shown here (Fig. 7; Additional file 1: Figures S22–S28) as total protein expression is significantly reduced (Additional file 1: Table S6). The expression of all these BBB transporters would also indicate that a capillary enriched sample had been assessed.

### Medication history of the cases

Additional file 1: Table S3 shows the medication history of the cases that have been used in this study. Sedatives, antidepressants and antipsychotic drugs (including haloperidol) were identified and listed together. A separate column lists all other medications.

## Discussion

This study aimed to answer an important clinical question using an integrative approach to investigate the interaction between two drugs and BBB transporters and their potential pharmacodynamic relevance in AD. The in vitro cell culture models and in silico computational model allowed us to [1] identify the transporters, [2] assess the potential mechanisms of amisulpride and haloperidol transport [3] perform studies on human and mouse brain endothelium and [4] minimize animal studies in line with the 3Rs (replacement, refinement and reduction principles). The in situ brain perfusion technique allowed us to [1] study the whole animal [2] and utilize a mouse model of AD. Importantly this is the only model to exhibit both amyloid-β_40_ and _42_ and tau pathology, mimicking human AD [39, 43]. Both plaque and tangle pathology are mainly restricted to the hippocampus, amygdala and cerebral cortex. Transporter expression in capillaries isolated from AD and age-matched human cases and mouse brain were also assessed. Medication history of the cases was evaluated.

Cell culture studies revealed a slow accumulation of [^3^H]amisulpride indicating a low BBB permeability. This can be linked to its low lipophilicity, as measured by the octanol-saline partition coefficient, and its inability to interact strongly with neutral and negatively charged lipid model systems thus its limited ability to passively diffuse across the lipid bilayer [22, 44]. Please note the plasma half-life of a single oral dose of amisulpride (50 or 200 mg) is ~12 h, which suggests that amisulpride will not have significantly degraded within the 2 h incubation period used in our study [45]. Furthermore the accumulation buffer does not contain plasma enzymes which would further increase the half-life of amisulpride in our cell culture models. Unlabelled amisulpride (20 µM) significantly reduced [^3^H]amisulpride accumulation in both cell lines, suggesting that there is a low affinity influx transporter at the plasma membrane or possibly at an intracellular organelle membrane (e.g. lysozyme) [46]. OCT1, OCT2 and 3 have been shown to be expressed in hCMEC/D3 and b.End3 cells in an earlier study by our group [32] and OCTN1 and OCTN2 were shown to be expressed in both cell lines in this present study. However, a study by Ohtsuki and colleagues found that the protein expression of OCT1, OCT2, OCT3, OCTN1 and OCTN2 were below detectable limits in hCMEC/D3 cells [47].

In line with the findings of [14] and our molecular docking studies, our accumulation studies suggested that OCT1 may be the influx transporter involved, as prazosin (substrate for OCT1 and 3) reduced [^3^H]amisulpride uptake in hCMEC/D3 cells after 2 h, whereas corticosterone (OCT3 substrate) had no impact on [^3^H]amisulpride accumulation in either cell line. In addition, OCTN1 and 2 inhibitors did not affect accumulation of [^3^H]amisulpride into hCMEC/D3 or bEnd.3 cells. However, in the presence of amantadine, a substrate for several transporters of organic cations (OCT1, OCT2 [48]), MATE 1, MATE 2 [49, 50] and PMAT [51]) there was no effect on hCMEC/D3 cells, and an increase in [^3^H]amisulpride accumulation in bEnd.3 cells after 2 h incubation, which suggests the involvement of an efflux transporter. This is consistent with previous observations [22], which found higher amisulpride transport in the basolateral to apical direction (Pe 5.2 ± 3.6 × 10^−6^cm/s) compared to the apical to basolateral direction (Pe < 10^−7^cm/s) in porcine brain microvessel endothelium. The differences in the effect of inhibitors on the two cell lines may be explained by amisulpride being a substrate for multiple transporters and variations in the function/expression of OCT1, MATE and PMAT transporters possibly related to species differences [52, 53]. The absence of any effect of prazosin on [^3^H]amisulpride uptake in bEnd.3 cells can be explained by prazosin-associated toxicity in this cell line, which causes protein values to decrease over the course of the experiment, resulting in no net effect on V_D_ values [32]. It is unlikely that the efflux transporter identified in our in vitro study is an ABC transporter, as neither ATP-depletion or substrates for P-gp, BCRP, or the MRP family had an effect on [^3^H]amisulpride accumulation in either cell line. Another study also revealed that amisulpride did not inhibit P-gp in an in vitro efflux assay [23], although they also utilised P-gp knockout and WT mice and these in vivo distribution studies suggested it was a P-gp substrate. Interestingly they considered that this may have little therapeutic impact due to its unusual receptor profile. Our single transporter computational studies revealed that amisulpride was not a P-gp substrate, but was a substrate for the SLC efflux transporters, PMAT and MATE1. Our in vitro hCMEC/D3 inhibitor studies with lopinavir, also indicated that amisulpride could be effluxed by PMAT. PMAT is thought to use a proton gradient to drive organic cation efflux from cells [53] and we (and others) have found that PMAT protein is expressed on human and mouse brain capillaries (Fig. 7, Additional file 1: Figures S19B, C and S28) [52–55]. PMAT mRNA has also been identified on the luminal and abluminal membrane of human, mouse and rat brain endothelial cells [53]. Other studies could not detect PMAT protein in isolated human capillaries and hCMEC/D3 cells [47, 56].

Interestingly our in vitro studies with the MATE1 inhibitor, famotidine, did not support the in silico data set which indicated that amisulpride was a MATE1 substrate. It is important to highlight that the lack of MATE1 inhibitor effect may not be conclusive proof of a lack of substrate interaction with the MATE1 transporter. It may be that the transporter was not sufficiently expressed [47, 52, 55, 56], although we could detect MATE1 protein in both our two cell lines, mouse and human brain capillary samples. Other considerations are that the inhibitor may need to reach a therapeutic concentration within the cell to elicit a response as has been previously observed with MATE1 inhibitors [50], the substrate and the inhibitor may bind to different binding sites, the non-specificity of the inhibitor, and that amisulpride interacts with both influx (OCT) and other efflux (PMAT) transporters making a response difficult to detect [26, 53, 57].

Haloperidol has been observed to have a high degree of dopamine receptor (D2) occupancy within the brain at very low doses suggesting that haloperidol is very efficient at crossing the BBB. This is in agreement with our in vitro BBB data. Please note that the half-life of haloperidol has been reported to range 14.5–36.7 h (or up to 1.5 days) after a single oral dose so will not have been metabolized significantly over our 2 h incubation period [58]. Haloperidol may cross the BBB by passive diffusion, as supported by the relatively high octanol-saline partition coefficient of haloperidol (0.6678 ± 0.1278) compared to amisulpride (0.0422 ± 0.0045), or may involve transporters. The use of transporters by haloperidol is confirmed by the self-inhibition studies in both hCMEC/D3 and b.End3 cell lines. As haloperidol exists predominately (94.8%) as a positively charged drug at physiological pH (pKa is 8.66) the transporter is likely to be OCT, which is expressed at the BBB. This was confirmed in vitro using the OCT substrates, amantadine (OCT1 and 2) and prazosin (OCT1 and 3). We also investigated the involvement of ABC transporters in the transport of haloperidol. For this, ATP was depleted from the cells by incubating them with 10 mM 2-deoxy-d-glucose. No effects of ATP depletion were observed compared to control in either cell line suggesting that haloperidol is not a *substrate* for the ABC transporters P-gp, BCRP, or the MRP family at the BBB as previously observed [59, 60].

Radiolabelled amisulpride (6.5 nM) was also incubated with haloperidol (OCT1 substrate and P-gp *inhibitor*) [25, 61]. Radiolabeled amisulpride accumulation was not affected by haloperidol (40 μM) in either cell line. This may be the result of interactions with both OCT1 and P-gp, although our inhibitor and in silico studies do not suggest amisulpride is a substrate for P-gp. Conversely when radiolabeled haloperidol (10 nM) was incubated with unlabeled amisulpride (20 μM) there was a significant decrease in accumulation. Overall these results may slight reflect differences in the interaction of amisulpride and haloperidol with different transporter binding sites.

Further insight into the interaction of amisulpride and haloperidol with the transporters was obtained through the in silico computational studies, where amisulpride showed binding affinities towards the binding sites of the influx transporter, OCT1, as well as the efflux transporters, MATE1 and PMAT. The binding affinities of amisulpride towards the binding sites of these transporters showed comparable energies for OCT1 and MATE1 with molecular level interactions through hydrogen bonds and hydrophobic interactions with a number of amino acids within the binding pocket. The nature of the interaction of amisulpride with OCT1 is similar to that observed for haloperidol and prazosin which are known substrates for this transporter. The binding affinity and level of interaction were slightly weaker with PMAT compared to OCT1 and MATE1, but still considerable level of interactions were observed suggesting amisulpride is a weak substrate of PMAT. Haloperidol showed comparable affinity for both MATE1 and PMAT suggesting it is a substrate for both transporters. MATE1 and PMAT are known to use a proton gradient to drive organic cation efflux from cells [53, 54, 62, 63]. MATE1, like P-gp, is thought to be expressed on the luminal membrane of the BBB, although this remains to be confirmed [64].

The in silico study supports the experimental observations and provides further evidence that amisulpride might be influxed through OCT1 and effluxed through PMAT and MATE1 but not P-gp. When amisulpride transport was investigated in vivo, a low BBB permeability to [^3^H]amisulpride was also observed in the wild-type mice and this did not change with age. Further studies in the 3×TG AD mice revealed an increased CNS uptake which was not accounted for by altered BBB integrity or changes in vascular space in the AD model mice, as there were no differences in [^14^C]sucrose uptake between the two groups; and neither was it explained by non-expression of the transporters studied (P-gp, OCT1, OCT2, OCT3, OCTN1, OCTN2, MATE1, MATE2 and PMAT). However, in post-mortem human brains expression of the efflux transporter MATE1 was lower in individuals with AD compared to age-matched healthy controls in the putamen; and PMAT showed a similar trend in the caudate nucleus, but there was no change in expression levels of these transporters in the frontal cortex. Importantly it has been reported that BBB impairments stem from AD abnormality instead of from vascular comorbidities [65]. Further evidence for regional tissue changes with AD came from our total protein measures (Additional file 1: Table S4). This may be linked to changes in the expression of other transporters such as P-gp [18] and GLUT1 [66] as well as the other SLC transporters measured in this present study. Interestingly regional differences are known to exist in the BBB transport and the intracellular distribution of antipsychotics [67]. The reduced MATE1 and PMAT expression observed in AD may therefore underpin the heightened sensitivity to amisulpride observed in the clinical population especially as together our in silico and in vitro studies suggested that amisulpride was a substrate for MATE 1 and PMAT. Several medications listed in Additional file 1: Table S3 (e.g. citalopram, metoclopramide and loratadine) have previously been identified as OCT1 inhibitors [25, 26]. It is not yet known if they also interact with MATE1 and PMAT. One of the medications (ranitidine) is an inhibitor of both OCT1 and MATE [64].

## Conclusions

This study included a detailed evaluation of transporter expression and usage at the BBB using in silico computational approaches, in vitro models and an in vivo animal model of AD as well as patient material. The datasets have provided evidence of an interaction of amisulpride and haloperidol with both influx (OCT1) and efflux (MATE1 and PMAT) transporters, which may be expressed at the luminal or abluminal membranes of the BBB and/or at an intracellular membrane. In vitro and in silico studies indicated that amisulpride was not a substrate for ABC transporters including P-gp. Furthermore, the study is of key importance as the results suggest that the heightened sensitivity to amisulpride observed in older people with AD is possibly due to previously unreported changes in SLC transporter expression, which increase amisulpride entry into, or possibly reduce clearance from the brain. This study is also the first step in the process of characterising age and AD-specific changes in SLC transporters of organic cations. Overall our study has implications beyond amisulpride prescribing, as it suggests that dose adjustments may be required for other drugs (e.g. haloperidol) which are substrates for SLC transporters in particular MATE1 and PMAT.

## Supplementary information

**Additional file 1.** Additional figures and tables.

## Data Availability

See Additional file 1.

## Abbreviations

OCT: organic cation transporters
OCTN: organic cation transporters novel
hCMEC/D3: immortalized human cerebral microvessel endothelial cell line
MATEs: multi-drug and toxic compound extrusion proteins
PMAT: plasma membrane monoamine transporter
3×TgAD: triple transgenic AD model
FBS: foetal bovine serum
BBB: blood–brain barrier
AD: Alzheimer’s disease
P-gp: P-glycoprotein
BCRP: breast cancer resistance protein
MRP: multi-drug resistance protein
RIPA: radio-immunoprecipitation assay
SLC: solute carrier
bEnd.3: mouse brain endothelial cell lines
VLOSLP: very late-onset (> 60 years) schizophrenia-like psychosis
NHS: national health service
MTT: 3-(4,5-dimethylthiazol-2-yl)-2,5-diphenyltetrazolium bromide
APP: amyloid precursor protein
PBS-T: PBS-TWEEN^®^ tablets
RT: room temperature
BDR: brains for dementia research
V_d_: volume of distribution
WB: Western blot
